# A framework for evaluating the costs of malaria elimination interventions: an application to reactive case detection in Southern Province of Zambia, 2014

**DOI:** 10.1186/s12936-016-1457-5

**Published:** 2016-08-11

**Authors:** Bruce A. Larson, Thandiwe Ngoma, Kafula Silumbe, Marie-Reine I. Rutagwera, Busiku Hamainza, Anna M. Winters, John M. Miller, Callie A. Scott

**Affiliations:** 1 Department of Global Health, Boston University School of Public Health, 801 Massachusetts Avenue, Boston, MA 80211 USA; 2 PATH Malaria Control and Elimination Partnership in Africa (MACEPA), National Malaria Control Centre, Chainama Hospital College Grounds, Great East Road, Lusaka, Zambia; 3 Ministry of Health, National Malaria Control Centre, Chainama Hospital College Grounds, Great East Road, P.O. Box 32509, Lusaka, Zambia; 4 Akros, Cresta Golfview Grounds, Great East Road, Unit 5, Lusaka, Zambia; 5 University of Montana School of Public and Community Health Sciences, Missoula, MT USA; 6 PATH Malaria Control and Elimination Partnership in Africa (MACEPA), 2201 Westlake Avenue, Suite 200, Seattle, WA 98121 USA

**Keywords:** Malaria, Elimination, Reactive case detection, Community case management, Cost, Zambia

## Abstract

**Background:**

This paper summarizes a framework for evaluating the costs of malaria elimination interventions and applies this approach to one key component of the elimination strategy—reactive case detection (RCD)—implemented through 173 health facilities across 10 districts in Southern Province of Zambia during 2014.

**Methods:**

The primary unit of analysis is the health facility catchment area (HFCA). A five-step approach was followed to estimate implementation costs: organize preliminary information; estimate basic unit costs; estimate activity unit costs; estimate and organize final unit cost database; and create the final costing database (one row of data per HFCA). By working through a specific application, the overall logic of the analysis and details of each step are presented. An electronic annex also provides all details of the analysis. Because population varies substantially across HFCAs, all results are reported per 1000 population in HFCAs.

**Results:**

During 2014, 38.9 households per HFCA were visited for RCD services; 166.8 individuals were tested and 32.3 tested positive and were treated. The mean annual cost per HFCA was $1177 (median = $923, IQR $651–$1417). Variation in costs was driven by the number of CHWs and passive cases detected. CHW-related costs and data review meetings accounted for the largest share of costs. Rapid diagnostic tests and drugs accounted for less than 10 % of total costs.

**Conclusions:**

The framework presented here follows standard methods in applied costing of public health interventions (combining ingredients- and activity-based costing approaches into one final cost analysis). Through an application to a specific programme implemented in Zambia in 2014, the details of how to apply such methods to an actual programme are presented. Such details are not typically presented in existing costing analyses but are required for applied analysts working with national malaria control programmes and other organizations to complete such analyses as part of routine programme implementation. Obtaining data and information for implementing the approach remains complicated, in part because analysts from one organization may not have easy access to information from another organization. This basic approach is transparent and easily applied to other malaria elimination interventions being implemented in sub-Saharan Africa and elsewhere.

**Electronic supplementary material:**

The online version of this article (doi:10.1186/s12936-016-1457-5) contains supplementary material, which is available to authorized users.

## Background

The Zambia National Malaria Control Centre (NMCC), in collaboration with partners, is supporting the implementation and evaluation of interventions designed to eliminate malaria throughout the country. These interventions include the distribution of long-lasting insecticidal nets (LLIN), indoor residual spraying (IRS), and intermittent preventive treatment (IPT) as well as more intensified strategies for community-level surveillance and population-wide and focal approaches to clearing parasites from people. Evaluating the costs of implementing such interventions has also been a component of overall evaluation activities.

Given the heterogeneity in both malaria transmission and implementation of elimination interventions across countries and regions within countries, the effectiveness of interventions are likely to vary depending on the implementation context [1–3]. Many of the same factors that influence effectiveness will also affect the costs of implementing the interventions.

For economic evaluations of malaria interventions in general, and cost-effectiveness analyses in particular, two essentially separate sets of studies or analyses needed to be completed: (1) the impacts of the intervention need to be estimated (for example, improved IRS coverage leads to reduced malaria incidence over a certain time period) and then translated into a common effectiveness metric (e.g., avoided disability-adjusted life years lost); and (2) the costs of the intervention/programme as implemented need to be estimated.

Studies reporting on the effectiveness of malaria interventions use a wide range of analyses, assumptions, and modelling strategies to evaluate effectiveness (see for example the literature review in [4]). Not surprisingly, when evaluating intervention costs, substantial heterogeneity also exists in costing analyses: detailed study-based costing analyses that follow or are part of randomized trials; costs extrapolated from information obtained from government health staff or offices as part of larger scale programmes (interventions implemented outside of a study setting); and costs estimated or created by modelling studies combining data from different sources (e.g., WHO CHOICE at [5]) For example, see White et al. [6] for a literature review that included 55 published studies that report on malaria intervention costs.

Although the concepts and basic methods for estimating the costs of implementing interventions are well documented [7–11], and recommendations exist for reporting on results [12], in practice such materials are relatively vague on details required for applying the methods to a specific programme/intervention and the multiple sets of preliminary analyses that typically need to be completed and then combined into one overall analysis of costs.

The cost of implementing a public health programme, whether for malaria elimination or any other objective, follows from the inputs used to implement the programme, where the programme transforms inputs into health outcomes. The cost of implementing the programme, conceptually, is just the sum of the costs of the inputs used to implement the programme, just like a receipt from a store lists the quantities of various items purchased and the price per unit. While the idea is simple, completing such an analysis requires making several decisions and completing several sub-analyses along the way.

Given that the cost effectiveness of interventions depends as much on the cost estimates as the effectiveness estimates, as well as the heterogeneity of costs and effects across time and space, it is surprising at times how little information is reported in the peer-reviewed literature on the detailed analyses and processes followed to estimate the cost of implementing malaria interventions or programmes. For example, the ‘costing’ section may include a brief summary of the generic methods prior to reporting results [13, 14] or delegate the costing methods to an appendix that also quickly summarizes the analysis but provides few practical details [15]. This lack of practical detail, driven by journal requirements for brevity, makes it difficult for academic as well as non-academic researchers, such as national malaria control programme staff or local NGO staff, to apply similar methods for their own programme activities and to understand how and why results vary across location and time.

The PATH Malaria Control and Elimination Partnership in Africa (MACEPA), in collaboration with national malaria programmes and other partners in Zambia, Ethiopia, and Senegal, recently completed a programme of evaluation research to estimate the costs of various malaria interventions implemented in regions of these countries during 2014. Interventions included, for example, the distribution of LLINs, IRS, and new strategies for rapid surveillance and population-wide and focal approaches to clearing parasites from people. Given the multiple countries, organizations, and interventions included in this programme of evaluation research, the cost-evaluation team developed a standardized framework/approach that was consistently applied across countries and interventions, while recognizing the heterogeneity inherent in the analysis due to variability in the implementation context.

The primary objective of this paper is to present in detail the basic framework the MACEPA team applied for evaluating the costs of these malaria interventions. To work through the necessary details, an application is presented for community malaria case management with reactive case detection (RCD), which is one component of malaria elimination strategies. This framework highlights the processes followed and the multiple sub-analyses completed as part of an overall analysis of implementation costs. This framework also identifies what drives costs along main steps in the implementation process. Such information is crucial for understanding the resources required to maintain, scale up, and replicate effective interventions in new settings and for identifying opportunities to adjust implementation strategies to reduce costs.

## An overview of the costing framework

The approach summarized below follows standard methods and guidelines [7–12, 16, 17], combined with experience gained from prior applied costing analyses (see [18–32]). Core components of the costing approach are summarized briefly below. The approach is then applied in detail to a specific example—estimating the costs of implementing community malaria case management with RCD during 2014 in Southern Province of Zambia.

## Geographic unit of analysis

In general, Ministries of Health (i.e., the NMCC in Zambia) and partners jointly work together to implement malaria interventions. In Zambia, various implementation activities occur at the national, provincial and district levels. Within districts, activities are often implemented with, or in relation to, a health facility targeting the population around that health facility, called the health facility’s catchment area (HFCA). Once a decision at the national level has been made to implement an intervention, typically in collaboration with provincial and district levels of government, and planning and other preparation work are complete, interventions are typically implemented and services delivered at the district level and often at the HFCA level. An HFCA unit of analysis for a costing analysis is logical, therefore, because substantial variation can exist across health facility catchment areas due to population size, acceptance of the intervention by local communities, geography, malaria prevalence, and other factors.

## Time horizon

In general, multiple malaria interventions and surveillance systems are implemented within the same catchment area over the same time period. For example, community malaria case management with RCD activities is an ongoing programme throughout a year. IRS and LLIN distribution are implemented in a catchment area in different discrete periods of time during an annual cycle. Population-wide and focal mass drug administration (MDA/fMDA) could involve two or more rounds of activities, but the goal is generally to complete the rounds within one yearly cycle corresponding to dry and wet seasons. Thus, the approach followed here estimates the costs of implementation per HFCA over a 12-month implementation period.

## Perspective

Incremental costs from the providers’ perspective are evaluated, recognizing that many providers/organizations collaborate to implement malaria interventions in a given country, including ministries of health, bilateral agencies, local and international NGOs, and other partners (such as UNICEF, WHO, etc.).

As noted above in the discussion of the geographic unit of analysis, on-the-ground implementation (e.g., RCD provided by community health workers or mass-drug administration campaigns) occurs within districts, and specifically within HFCAs inside a district. Because on-the-ground implementation occurs at the district level, the focus is on costs for inputs physically used for implementation activities within districts in Zambia, or an equivalent level in other countries.

Support for implementation comes from other levels of the health system down to each district, and HFCAs within districts, in multiple forms including training activities, the delivery of supplies, and site visits for supervision conducted by staff from the district health office or partner agencies. As an example, training of trainers (TOT) is one common activity that often occurs prior to implementation activities. TOT activities are typically organized in one location in a province, for example, and a limited number of staff members from various districts in the province are trained at the same time. These district-based staff members are then the trainers that provide training services within a district to many individuals (e.g., community health workers) involved with on-the-ground implementation across many HFCAs in the district. Even though the TOT occurs at the provincial level, the trainers then work within districts, so they are directly (physically) involved at the district level with implementation activities. So TOT costs are included in the analysis.

Given the focus on the costs of inputs (resources) directly used for implementation activities within districts, costs to providers for resources used above the district level, such as national planning meetings, preparation time for individual organizations involved in such meetings, are excluded for this analysis. Estimating “higher level” costs has proven difficult in large part because of the number of activities (often meetings and procurements addressing elimination activities but also other topics) and organizations involved. In addition, costs to the health system that would be incurred regardless of whether a specific malaria intervention or surveillance system was implemented are also excluded. In sum, the goal is to estimate incremental costs to the providers directly used at the district level and below (HFCA in Zambia).

Costs to households receiving intervention services (e.g., waiting time while IRS completed for a building, time meeting with community health workers during mass drug administration activities, potentially negative side effects of medications) are excluded from this analysis. Evaluating the costs (and benefits) to households is perhaps a useful activity, but requires a different type of analysis and data.

## Financial and economic costs

The term financial cost to a provider typically is used to denote actual payments incurred by that provider over a certain period of time, while economic cost generally refers to social opportunity costs (see [7, 8]). Economic costs can differ from financial costs for many reasons (tax policy, subsidies, donations, market imperfections, and partial equilibrium affects associated with large programmes). In the approach used here, the goal is generally to use and estimate economic costs.

In most applied costing analyses for health interventions, three main issues create a distinction between financial and economic costs: (1) capital purchases (e.g., a vehicle) purchased in prior years or the programme year but available to be used over many years; (2) donated or heavily subsidized inputs (e.g., donations chemicals through a bilateral Aid programme) or conversely heavily taxed inputs; and (3) labour provided by individuals who are called volunteers who may or may not receive various types of payments (stipends, tokens of appreciate, daily subsistence allowances). Such issues, and how to address them, are highlighted in more detail in the application provided below.

In basic economic theory, inputs used in production processes are considered fixed or variable. Fixed inputs cannot be adjusted over a certain period of time, while variable inputs can be adjusted easily over time. Fixed inputs include *capital* inputs (e.g., equipment, buildings, vehicles) that last for more than 1 year [16]. It is standard practice to estimate an annualized equivalent cost, based on a real discount rate and expected equipment life, to translate the one-time payment into multiple annual equivalent payments (or monthly). This topic is addressed further below. Capital inputs might also be leased on a long-term contract (e.g., a building lease for 10 years), in which case an expense occurs regularly over time (e.g., monthly, yearly). Employees on long-term contract are also fixed inputs in many situations. Variable inputs are those inputs that can be adjusted easily over time, and variable costs are the associated costs for these inputs. Labour hired on short-term contract, quantities of fuel purchased, and quantities of drugs dispensed are typical examples of variable costs.

The distinction between recurring and non-recurring costs is not especially relevant for this costing framework. The goal is to estimate the annual cost of implementing an intervention (based on actually implementing the intervention). For analyses where cash flow over time is relevant, such as budgeting for a multi-year programme or running a business, a distinction is often made between recurring and non-recurring costs. Recurrent costs are costs organizations incur regularly over time (e.g., every month or year), which include the costs for variable inputs as well as fixed inputs (equipment, buildings, vehicles, staff) on long-term contract. While useful for understanding organizational cash flow, the distinction is irrelevant for understanding the annual economic cost of implementing an intervention.

## Costing approach—bottom up with ingredients-, expenditures-, and activity-based costing

A bottom-up, ingredients-based costing approach is used where the quantity of each type of input is multiplied by a price (unit cost) to estimate costs for that input, and then total costs are the sum of the costs for the various inputs. However, expenditures (quantity times price) are used at times depending on the specific input and availability of data. For example, an organization importing LLINs (such as UNICEF) typically will have a total expense for importing (and delivery to a specific location in a country). Depending on their contract for purchasing and importing the nets, costs might be broken down by the item (in this case LLINs) and then a combined total for any other associated charges (including importing fees, transportation). Typically, there is little reason to attempt to disaggregate the “other charges” further.

The cost of an intervention or surveillance system also depends on how it is implemented, which typically occurs through completing a series of activities (or steps, or stages). For example, with many programmes or interventions, training of trainers for the programme occurs first, and then these trainers providing training to, for example CHWs to implement RCD, and then the actual provision of intervention services begins in a health facility catchment area. When discrete activities occur as part of the overall intervention, the cost of the activity is estimated first (typically using both ingredients- and expenditure-based costing), and then the unit cost of the activity is included when completing the overall costing analysis. Linking costs to specific implementation activities is also useful for understanding how an intervention might be replicated in other locations and time periods as well as for assessing how costs might change if implementation activities were altered.

## Overview of analytic steps

**Fig. 1:**
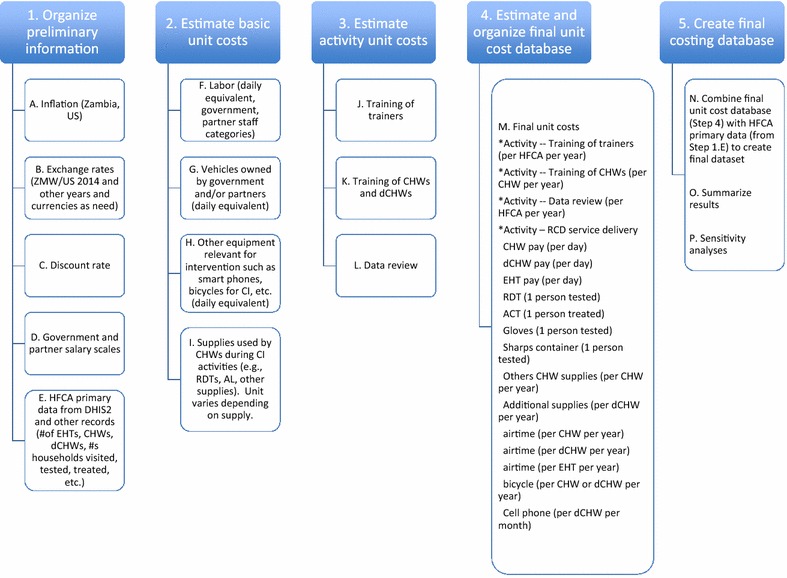
Five stages of the costing approach

Figure 1 provides an initial overview of five stages of information gathering and analyses that, when combined, comprise the overall costing framework. The five main stages are:

1. Organize preliminary information;
2. Estimate a set of basic unit costs;
3. Estimate an additional set of activity-specific unit costs, which may also use unit costs estimated in stage 2;
4. Estimate and organize a final ‘unit cost’ database; and
5. Combine the final unit cost database with HFCA-level programmatic implementation data (organized as part of stage 1) to create a final cost database (unit of observation is the HFCA).

## Detailed application to reactive case detection in Zambia

Malaria community case management with reactive case detection (denoted as RCD throughout this paper) consists of visiting the home of an incident malaria case detected passively, where passively detected means a person tests positive for malaria after presenting at a health facility or after seeking care from a CHW in their community [33]. For a patient diagnosed with malaria, based on a positive malaria rapid diagnostic test (RDT) result, a CHW then visits the patient’s home and offers a malaria RDT to additional household members and those found in surrounding households (active follow-up). Those individuals testing positive through active follow up are offered an anti-malarial treatment following national treatment guidelines. In Zambia, at the time of this analysis, information about passive and active follow up was recorded on paper registers by CHWs. Aggregate data over time (e.g., monthly) for each CHW were submitted directly to the National Malaria Control Center’s District Health Information System 2.0 (see [34]) by a CHW trained additionally on these processes (called a data CHWs) using a mobile phone.

The remainder of this section works through the five stages presented in Fig. 1 in detail. In addition, an additional Excel file is provided that documents all details of this analysis (Additional file 1). In the discussion below, reference to specific sheets within the RCD Workbook (Additional file 1) will be noted as, for example, “sheet—Zambia inflation”, to locate the specific worksheet in the overall file. In addition, Table 1 provides basic information used throughout the analysis (e.g., costing year, exchange rate, discount rate used).

**Table 1:** General information

Study location	Southern Province, Zambia
Number of districts included in analysis	10
Number of HFCAs included in final analysis	164
Geographic unit of analysis	Health facility catchment area
Time horizon for analysis	2014 calendar year
Perspective for costing analysis	Providers (Government, MACEPA)
Costing approach	Bottom up (ingredients, expenditures, activities), incremental costs
Main currency for analysis	Zambian Kwacha (ZMW)
Other currency included in analysis	US$
2014 annual average exchange rate (2014 ZMW/$)	6.39
Inflation (Zambia and US)	CPI, annual average
Real discount rate	3.00 %
Year for reporting costs	2014

## Organize preliminary information (stage 1)

A set of preliminary information is required to begin the analysis. For the analysis of RCD implementation costs, stage 1 consists of organizing five categories of preliminary information (items A–E in Fig. 1).

### A. Inflation

The analysis included RCD operations for 2014. Any costs incurred in Zambian Kwacha (ZMW) in years prior to 2014 for inputs used in 2014 were adjusted “up” for inflation using Zambia’s consumer price inflation data available through the International Monetary Fund (IMF) World Economic Outlook (WEO) Database [35]. In Additional file 1, see sheet—Zambia inflation and sheet—US inflation for specific numbers used in the RCD analysis. Some analysts adjust for inflation using the gross domestic product (GDP) deflator, which only considers inflation for domestically produced goods and services, rather than consumer price inflation, which also includes inflation through imports. Since the implementation of malaria interventions in Zambia, as well as other countries, involves some substantial amount of imported items, the CPI is used in this analysis. As shown in the Zambia inflation sheet in Additional file 1, the differences between the two numbers for Zambia are relatively minor, and the GDP deflator data (also as index number) are also available at the same IMF WEO database.

### B. Exchange rates

When resources used to implement an intervention are imported and/or purchased in another currency, an annual average exchange for the year of purchase is used (see Oanda.com for historical exchange rates) to convert to the relevant local currency. For example, the annual average exchange rate for 2014 between the Zambian Kwacha and the US$ was 6.39 ZMW/US$ (see Additional file 1, sheet—exchange rates). All primary analyses of costs are completed in local currency (ZMW for Zambia). For final reporting purposes, final results are reported in US dollars using the annual average ZMW/US$ 2014 exchange rate. As an example, if an input is purchased in Euro in 2013, the Kwacha/Euro exchange rate in 2013 is used to convert to the local currency and then inflated up to 2014 using Zambian inflation index numbers. The 2014 ZMW/US$ rate is then used for converting to US dollars in 2014 for final reporting.

### C. The discount rate

Conceptually, the discount rate used for evaluating government programmes should reflect the government’s opportunity cost of funds for public investments [7]. In practice, a 3 % real discount is commonly used in public health programme evaluation activities [8]. As a base case, a 3 % real discount rate is used. This discount rate is used mainly for estimating annual equivalent costs of equipment or other investments (such as certain types of training activities) used for implementing the programme in the year of analysis. In general, the malaria interventions are not equipment/capital intensive, and equipment such as vehicles, are often rented rather than purchased, so total costs are not especially sensitive to the choice of the discount.

For sensitivity analyses, the real cost of government long-term borrowing provides an alternative approach for estimating a real discount rate, which can be estimated using an annual yield on long-term bonds minus annual inflation [7]. For example, the yield on 10-year Zambia government bonds was 18.75 % for the May 2104 tender (see [36] for government bond information). Annual inflation in 2014 was 8 % (see WEO database referenced above), which suggests a 10.75 real cost of government borrowing in 2015.

### D. Salary scales for government and partner staff categories

Various categories or cadres of individuals contribute to implementing RCD in various ways. To begin, it is useful to obtain information on annual full costs to employers for key categories of such individuals who are hired as ‘staff’, typically on an annual or long contract. Full cost includes all forms of compensation (salary, benefits, allowance, etc.) along with expected work schedule (e.g., 5 days per week) along with various categories of leave (national holidays, annual leave, sick days, etc.). See Additional file 1, sheet—GRZ salary scale and sheet—partner salary scales. Rather than using the actual salary for a specific person (e.g., the actual salary for each specific malaria programme staff member in a specific district), a typical salary for that cadre of worker is used (e.g., median of steps within that grade). The details of this choice will vary across analyses depending on the information available.

### E. Primary data at the HFCA level on programme implementation

The HFCA is the primary unit of analysis for reporting on costs of RCD implementation. The Zambia DHIS2 was queried to obtain data on key programme inputs and outputs for each HFCA (reported on a monthly basis by data CHWs working with each health facility) for 2014. Key information includes the number of passive cases detected, the number of households visited, the number of people tested, and number treated. A total of 173 HFCAs across 10 districts implemented RCD activities in 2014. A small number of HFCAs began RCD activities during July or August of 2014. In this case, a simple projection was made to estimate a full year of implementation (e.g., if RCD begin in August 2014, the data for the 5 months was multiplied by 12/5 to project up to 12 months).

In addition, data for the number of CHWs, data CHWs, and environmental health technicians (who supervise RCD activities), and the population for each HFCA were required for estimating costs and obtained directly from the implementing organization(s). When this HFCA-level information is organized into one dataset, with one observation per HFCA, the first stage of the costing approach was complete (items 1–5 in stage 1 in Fig. 1 are complete). See Additional file 1 sheet—HFCA primary data for this dataset.

## Estimate basic unit costs (stage 2)

The purpose of stage 2 is to estimate a set of unit costs for various resources used during the implementing of RCD. In Fig. 1, four categories of ‘basic’ unit costs are labour, owned vehicles, other equipment, and supplies used by CHWs and data CHWs during RCD activities. The processes used to create these basic unit costs are summarized below (and results are provided in Table 2). Note that the “unit” for each unit cost is logically different at this stage of the analysis.

**Table 2:** Basic unit costs

	Unit	USD 2014
Provincial medical officer	Per day	108.57
NMCC programme officer	Per day	83.28
District medical officer (DMO)	Per day	79.48
District health office (DHO) staff, malaria focal person	Per day	53.50
Health facility supervisors (environmental health tech)	Per day	40.72
Community health workers	Per day	11.03
Driver (DHO)	Per day	32.50
MACEPA driver	Per day	48.93
MACEPA technical staff	Per day	175.92
Standard programme vehicle (owned by MACEPA or government)	Per day	79.43

### F. Labour (daily wage cost)

Three categories of labour typically contribute to implementing malaria interventions, including RCD: labour on annual salary (staff typically with a long-term employment contract); labour receiving a daily wage or other non-salary payment (but no long-term employment contract); and labour called volunteers (see Additional file 1 sheet—Unit costs labour for details).

For staff receiving an annual salary (salary scales obtained as part of stage 1.D), full annual costs (including salary, benefits, and any types of allowances or per diems) are divided by expected annual working days to estimate a daily salary cost. For example, with 365 days a year, and 7 days per week, there are 52.14 weeks per year. If an employee is expected to work 5 days per week, with 20 days of annual leave, 13 national holidays, and five sick days, the employee is expected to work 222.7 days per year. Thus, the full annual cost divided by 222.7 would be their salary per day or the unit cost of this type of labour. The cost for this type of labour is then the daily cost times the number of days working on the intervention during a year.

Other categories of workers, not technically ‘staff’, may receive a daily wage, stipend, allowance, or other non-salary payment (e.g., workers loading trucks, individuals spraying houses during IRS activities, etc.). Such payments could be per day, or per load, or per household depending on the intervention and payment (or per load, or per household visited).

In addition, many public health programmes rely on ‘volunteers’. The word volunteer is often used but not precisely defined. For example, CHWs in Zambia are called volunteers, but nonetheless receive various benefits from supporting the implementation of interventions and surveillance systems, including: daily subsistence allowances during trainings and cell phone top ups as part of RCD activities discussed later.

In the case of RCD implementation, CHWs perform the follow up activities with households as part of ‘routine’ activities (unlike campaign-type work with IRS, net distribution and mass drug administration). For the RCD costing analysis, a minimum government formal wage was used to develop an example daily wage for CHW labour for RCD activities (see Additional file 1 sheet—unit costs labour for details), which is used as the opportunity cost of labour when reporting economic costs.

### G. Owned vehicles (daily vehicle cost)

Vehicles owned by the government or partner organizations are typically used for implementing public health interventions, including RCD. Vehicles are a typical example of capital equipment that is purchased once but then used over multiple years. Vehicles are typically used during training activities (transporting staff to training venues) and during various other implementation activities (such as data review activities, etc.). The purpose at this point in the analysis is to estimate a monthly equivalent cost for typical vehicles used during RCD activities (based on the discount rate and expected vehicle service life).

Identifying the purchase price in the year of purchase if available, inflated up to the year of analysis (or the current market value of a similar item), is the first step in this process. In any spreadsheet programme, this monthly equivalent cost can then be easily estimated (see Additional file 1 sheet—unit costs vehicles for details). The annual discount rate is 3 % based on stage 1. For the life of equipment, the base assumption is 5 years for major equipment but shorter for less durable equipment.

Once the monthly equivalent cost for a vehicle is estimated, the additional standard costs of owning a vehicle are added—insurance, maintenance, any taxes or other fees such as license—to estimate a monthly equivalent cost for the owned vehicle. Once the monthly equivalent cost of a vehicle is estimated, the typical days of operation per month are used to then estimate a daily equivalent vehicle cost. With 20 typical working days per month on average (as accounting for national holidays), the base assumption is a vehicle can be used 19 days per month on average, assuming 1 day per month obtaining repairs, servicing (see Additional file 1 sheet—unit costs vehicles for details).

For this RCD analysis, costs associated with a recent project vehicle purchase as well as related recurring costs (maintenance, insurance, etc.) was used to estimate a standard daily cost equivalent for vehicles used by government or MACEPA staff to support RCD activities. While staff from multiple organizations, such as provincial and district officials, used vehicles from their organizations to contribute to implementation activities (e.g., travel to contribute to training, travel for supervision), details on such vehicles were not easily available or worth the effort to obtain.

Note that vehicles are often rented to support the implementation of interventions. In such cases, rental costs (per day, month, year, etc.) are used directly. If a driver was included in the rental cost for a vehicle, then both the vehicle and driver are included in the rental costs. It is often the case that records will not exist to separate just the vehicle cost from the driver costs.

### H. Other equipment

Cell phones for data CHWs used for reporting data and bicycles provided to CHWs to facilitate transport are the two other main types of equipment used for RCD activities. The same process as described above for vehicles in used to develop annual or monthly equivalent costs for any other equipment (i.e., items that provide services over more than 1 year). When completing this analysis, any additional procurement/transportation/distribution costs associated with purchasing the equipment and delivering to the user (e.g., CHWs for bicycles) are included in the upfront cost that is then annualized (see Additional file 1 sheet—unit costs Other Equip for details).

### I. Supplies used during RCD implementation

CHWs use RDTs to test for malaria and then treat those testing positive with an anti-malarial drug according to national treatment guidelines. In addition, CHWs receive and use a variety of other minor supplies during RCD activities (see Additional file 1 sheet—unit costs–RCD supplies). The unit cost of an RDT was estimated at $0.36, which was based on the unit cost incurred by MACEPA to purchase RDTs during 2014. This unit cost includes the RDT as well as all shipping, handling, and insurance.

In Zambia, individuals testing positive in 2014 were generally treated with artemether-lumefantrine (AL). While the numbers of individuals tested and treated are recorded and uploaded to the DHIS2 system, the details of the dosage provided to these individuals are not. Four standard courses of treatment exist based on 6, 12, 18 or 24 pills (1–4 pills per dose, two doses over 3 days). To estimate a standard unit cost of AL, the weighted average of all courses of AL dispensed in the districts in Southern Province, based on proportions of each dose dispensed, was estimated and used as a standard unit cost for AL. For reference, the cost for the standard adult course of 24 pills was $1.814 in 2014 (UNIMED cost), while the weighted average unit course across all doses was estimated at $1.35 (see Additional file 1 sheet—unit costs–supplies).

CHWs, data CHWs, and EHTs receive airtime sent directly to their phones monthly if information is recorded and uploaded to the DHIS2 system. In addition, CHWs receive and use a variety of other supplies (note books, gloves, sharps disposal containers, plastic bags) within their CHW kit. Based on estimates of quantities used for RCD activities, a standard “unit cost of other supplies per year per CHW” was estimated at $63.72. Several additional assumptions were needed to complete this estimate, with all details provided in Additional file 1 sheet—unit costs–supplies.

## Estimate activity unit costs (stage 3, J–L)

Prior to implementing RCD activities in an HFCA, CHWs and data CHWs complete a short training programme (a few days), with multiple training sessions provided across districts in the province. The trainers leading the CHW/data CHW training sessions also complete a short course (5 days) that was organized for the province. After implementation of RCD activities, data review meetings are also held at the district level with district health facility staff to review implementation progress, identify gaps and review data collectively.

### J–K. Training of trainers and CHWs

The processes used to estimate unit costs for training of trainers and training CHWs/data CHWs are identical. As an example, we focus here on estimating the unit cost of a CHW training. CHW trainings were provided at the district level. The structure of the trainings is very similar, and the cost of one training workshop (held in February 2013 in Mazabuka) was used to estimate a unit cost of CHW training (see Additional file 1 sheet—UC training CHWs for all details). Table 3 provides a summary of the information and additional analyses used to estimate a unit cost for training CHWs (unit is cost per year per CHW).

**Table 3:** Unit cost for CHW training

Input category	Resource	Unit	Total units	Unit price	Total cost (2013 ZMW)
DSA, non MACEPA	DSA CHWs	Day	90	300	27,000
	DSA HF supervisors	Day	9	500	4500
	DSA trainers (DMO staff)	Day	12	500	6000
	NMCC parasitologist	Day	4	600	2400
	NMCC driver	Day	4	350	1400
DSA for MACEPA	Accommodation	Day	15	300	4500
	M&IE	Day	20	210	4200
Other allowances	Lunch allowance	Day	12	50	600
Transport	Transport allowance	Day	12	100	1200
	CHW/other transport allowance	Day	66	50	3300
	Bus hire for field work	Bus trip	2	1150	2300
	MACEPA vehicle	Vehicle days	4	508	2031
	Fuel for MACEPA vehicle	Total fuel			200
	GRZ vehicle	Vehicle days	4	508	2031
	Fuel for NMCC vehicle	Total fuel			200
Venue costs	Venue	Day	2	300	600
Refreshments	Mineral water	Portion	100	3	300
	Snacks	Portion	100	15	1500
Other costs	Bostik	Total			8
	Printing and copying	Total			300
	Airtime	Total			100
	Sim cards and airtime	Total			160
	Renting storage space	Day	4	120	480
Stationary and printing	Stationary	Total			1527
	Printed materials	Total	36	28	704
Staff salaries	Environmental health officer	Day	12	260	3123
	Midwife nurse	Day	12	260	3123
	DMO pharmacist	Day	3	508	1524
	MFPP/EHT	Day	3	260	781
	District trainers	Day	12	342	4102
	NMCC parasitologist	Day	4	508	2032
	NMCC driver	Day	4	208	831
	MACEPA technical	Day	8	1124	8994
	MACEPA support	Day	4	313	1251
	MACEPA PADM	Day	4	313	1251
	MACEPA driver	Day	4	313	1251
Total (2013 ZMW)					95,802
Total (2014 ZMW)	(8 % inflation 2013–2014)				103,466
Total (2014 USD)					$16,190
Number of CHWs trained					30
Cost per CHW					$540
Annualized cost per CHW (2014 USD)	(3 %, 3 years)				$185

Training activities typically have a similar structure, with a few main categories of inputs used (and therefore costs) during the training sessions. Key categories are discussed below.

#### Daily subsistence allowances

Since participants in training activities (both trainers and trainees) typically need to travel to the training locations. A daily subsistence allowance (DSA) is typically provided to participants to cover meals and lodging. DSA expenditures (based on daily rates and number of individuals) is a key category of costs of training activities.

#### Transportation

In addition, trainings typically require that individuals travel to the training location. In addition to DSA, a separate transport allowance was provided to CHWs and their trainers. As part of the training activity, a bus was also hired for fieldwork (RCD practice in the community).

Other individuals participating in trainings travelled with their own vehicles and drivers, such as staff from the National Malaria Control Center (their organization’s vehicle). Although a fuel allowance was included in the budget for the training, the basic cost for the use of the vehicle was not. As a result, the unit cost per day for vehicle use (estimated in Step 2.G) times the number of days used for the training was used to include a vehicle cost into the analysis.

Transportation costs are a good example where ingredients and expenditures are both used for estimating costs. For example, an ingredients-based approach was used to estimate the cost for a vehicle used by government staff to attend a training course (days of use multiplied by an average daily vehicle cost). The use of this vehicle does not show up typically as a ‘training expense’ because the government already purchased the vehicle. However, the bus rental (based on a rental cost per day) was based on total expenditures (including the vehicle, driver, fuel, insurance). Here, an expenditure approach for the bus is adequate (disaggregating further into components—just the vehicle, just fuel, just driver—is not needed).

#### Venue costs and other minor supplies

These other inputs include meeting space for trainings, stationary, printing and copying, and other supplies used during the training sessions.

#### Labour

Labour is an obvious input into training activities. The trainers and others in supervisory roles ‘provide’ the training, while the trainees (e.g., CHWs) ‘receive’ the training. For CHW training, the district trainers (who received prior training) along with other staff at the district, provincial, and national level attended/worked at CHW training sessions. In addition, drivers were also used by these staff (with their vehicles discussed above) during the CHW trainings. Salary-related costs for staff attending trainings are calculated as the daily salary equivalent times the number of days participating. Because their salaries were paid by their organization, such costs do not show up in a training programme budget/expenditures but are a cost of implementation.

#### Final unit cost

The estimated cost for one CHW training session was ZMW 95,802 (in February 2013). This cost was then inflated up to 2014, for a total of ZMW 103,467. With 30 CHWs trained, the cost per CHW is estimated at ZMW 3449 ($540 per CHW).

Training for RCD activities is essentially an investment in human capital, so that the same process used to estimate an annual equivalent cost for owning a vehicle is followed to estimate an annual equivalent cost for training. As a base case, we assume that training is required every 3 years for RCD (either a CHW is no longer working with the health facility so a new CHW would need training or refresher training would be needed after 3 years). With a three-year service life for the training and a 3 % discount rate, the annual cost for CHW training is estimated at ZMW 1184 (or $185 per CHW per year).

### L. Data review

Data review meetings are held within districts, which were attended by one person per health facility (the dCHWs sending data up to the DHIS2 system) as well as district and provincial staff and MACEPA staff. The cost for a data review meeting per district (one per year) is estimated following the same logic as for training sessions (see Additional file 1 sheet—UC data review).

## Organize final unit cost database (stage 4, M.)

The information gathering and analyses completed as part of stage 1–3 generate a set of ‘final’ unit costs that are used for the last stage of the costing analysis (combining a set of unit costs with quantities of resources used). For the RCD analysis, this final set of unit costs is provided in Table 4 (also in Additional file 1 sheet—summary final unit costs). The four main categories of ‘final unit costs’ are: training; daily pay; supplies; and equipment. These are called “final unit costs” because they are combined with the HFCA primary data for completing the costing analysis at the HFCA level over one year (2014 in this analysis). Thus, all final unit costs must be in a unit that, when combined with the primary HFCA data, leads to a cost at the HFCA level.

**Table 4:** Final unit costs

	Unit	USD 2014
Training of trainers (per HFCA per year)	Per HFCA per year	38.94
Training of CHWs (per CHW per year)	Per CHW per year	185.23
Data review (per HFCA per year)	Per HFCA per year	813.42
CHW pay (per day)	Per day	11.03
dCHW pay (per day)	Per day	11.03
EHT pay (per day)	Per day	40.72
RDT (1 person tested)	1 patient tested	0.36
Gloves (1 person tested)	1 patient tested	0.06
Sharps container (1 person tested)	1 patient tested	0.12
ACT (1 person treated)	1 patient treated	1.34
Others CHW supplies (per CHW per year)	Per CHW per year	63.72
Additional supplies (per dCHW per year)	Per data CHW per year	1.10
Airtime (per CHW per year)	Per CHW per year	33.80
Airtime (per dCHW per year)	Per data CHW per year	46.85
Airtime (per EHT per year)	Per EHT per year	11.71
Bicycle (per CHW or dCHW per year)	Per CHW or dCHW per year	29.53
Cell phone (per dCHW per month)	Per dCHW per year	31.84

For example, the ‘unit’ for training of trainers and data review meetings is a cost per HFCA per year, while the ‘unit’ for CHW training is a cost per CHW per year. All other final unit costs are based on either: (1) costs per CHW, dCHW, or EHT per year in the HFCA; or (2) costs per person tested or treated.

## Create final costing database (stage 5)

The final stage of the analysis is to combine the HFCA primary dataset and the final unit cost dataset to estimate costs per HFCA per year. Depending upon the information available from stages 1–4, additional variables may need to be created at this stage to complete the analysis.

### N. Create final dataset

This step combines the HFCA primary dataset with the final unit costs to estimate total costs. While this analysis could be completed in a number of different software packages, STATA was used in this analysis to complete stage 5. After importing the HFCA primary data into a STATA dataset, a STATA do file was written to import final unit costs and complete additional calculations (see Additional file 1 sheet—Do file).

The STATA do file first creates variables for the primary unit costs and specifies their values (from Table 4). In the final set of unit costs, salary costs per day for CHWs, dCHWs, and EHTs are included, but data in the HFCA primary data set only include the number of these types of workers. No records exist to document the level of effort for individual CHWs, dCHWs, or EHTs allocated to RCD activities. Based on informal conversations with programme staff and health facility staff, the following additional assumptions were used: EHTs allocated 4 h (0.5 days) per month for supervising RCD activities; dCHWs allocated 2 h per week for organizing data and reporting up to the DHIS2 system (0.25 days per week). CHW levels of effort for providing RCD services are based on the number of households visited (assumption of 1 h per visit) and additional time per person tested (15 min per person).

The next step is to multiply unit costs by the appropriate quantity in the dataset to estimate a cost for that input/resource. For example, the cost of training per year in an HFCA is the unit cost for training of trainers (a cost at the HFCA level) plus the unit cost of training a CHW times the number of CHWs providing RCD services. The cost of RDTs is the unit cost per RDT times the active number tested, while the cost for AL is the unit cost for AL times the active number treated. This process is completed for each final unit cost and related quantity in the dataset. The end result is a total cost for each category of resources used for RCD, and the total costs are just the sum of the individual components of costs.

The population served by the health facility (the HFCA population) varies substantially across HFCA (from under 1000 to over 20,000). As a final step, costs per HFCA are divided by the population of the HFCA (in 1000s) to create a population-standardized cost (cost per 1000 population per HFCA).

### O. Summary results

Basic summary results—mean, median, and interquartile range—are reported in Table 5. Key variables that will drive cost estimates per 1000 in the population are reported first. While the final data set has 173 observations (HFCAs), HFCA population data did not exist for nine HFCAs. The average HFCA had a population (in 1000s) of about six (median = 4.9; IQR 3.0–7.8), although a few have small populations (<1) and a few have very large populations (>14). For the 164 HFCAs with population data, HFCAs had on average 1.5 CHWs who visited 38.9 homes, tested 166 and treated 32.3 individuals per 1000 population. The distribution of the number of homes visited and individuals tested and treated are all skewed so that the medians are substantially less than the means. These basic variables—population, number of CHWs, active households visited, number tested and treated—drive overall costs per HFCA.

**Table 5:** Basic health facility catchment area results for RCD during 2014

Variable (n = 164)	Mean	Median	p25^a^	p75^a^
HFCA population (1000s) in 2014	6.1	4.9	3.0	7.8
Number of CHWs per HFCA	1.5	1.3	0.8	1.8
Active households visited per HFCA	38.9	17.5	8.1	45.3
Active number of individuals tested per HFCA	166.8	89.2	32.1	193.6
Active number of individuals treated per HFCA	32.3	4.3	0.9	17.1
Total cost per HFCA	1177	923	651	1417
Activities				
Cost of training (training of trainers and CHWs) per HFCA	347	285	193	406
Cost of data review per HFCA	225	165	104	269
Cost for RCD service delivery (implementation) per HFCA, comprised of:				
Cost of CHW bicycles per HFCA	54	44	30	63
Cost of data CHW cell phones per HFCA	10	8	5	12
Cost (salary) for environmental health technicians (EHTs supervise CHWs) per HFCA	68	50	31	81
Cost (imputed salary) for CHWs per HFCA	111	54	22	129
Cost (imputed salary) for data CHWS per HFCA	45	34	21	53
Cost of RDTs per HFCA	60	32	12	70
Cost of AL per HFCA	43	6	1	23
Cost of CHW supplies (gloves and sharp containers) per HFCA	30	16	6	35
Cost of other CHW supplies per HFCA	116	95	64	136
Cost of other data CHW supplies per HFCA	0.35	0.26	0.16	0.41
Cost for CHW airtime per HFCA	51	43	28	60
Cost for data CHW airtime per HFCA	15	11	7	17
Cost for EHT airtime per HFCA	3	2	2	4

In Table 5, all costs are reported in 2014 USD, the HFCA is the unit of analysis, and all variables reported per 1000 HFCA population

^a^p25 and p75 are the 25th and 75th percentile of the distribution

The mean annual cost per 1000 population across the 164 HFCAs is $1177 (median = $923, IQR $651–$1417). HFCAs with high total costs per 1000 population (>75th percentile, n = 41) are generally HFCAs will smaller population than average (2.6) and a relatively large number of CHWs for the population (2.7 per 1000 population). The largest components of total costs are training, data review, and CHW time and supplies. Drugs and diagnostic tests are a relatively minor share of total costs.

Community case management with RCD is transmission dependent and serves somewhat like an insurance role in an overall package of malaria elimination interventions; a certain set of costs are incurred even if a small number of households are visited by CHWs, which would be the case if few malaria cases were passively detected in the health facility or community. For example, from Table 5, if even 0 households were visited, average costs per HFCA would remain at about $850 per 1000 population (eliminate CHW labour time and associated supplies, RDTs, drugs, etc.).

As with most malaria interventions, their need and how they are implemented is likely to vary depending on transmission. The scale and costs associated with community case management with RCD in Southern province presented here were related to the level of transmission at the time of rolling out these activities. If the number of passive cases detected in the HFCA were substantially smaller across most HFCAs, scaling back or changing the method of deploying these activities might occur. This emphasizes the point made earlier that costs depend on how an intervention is implemented.

Costs per programmatic output (number houses visited, individuals tested, individuals treated) are often reported in costing analyses and the numbers are easy to calculation. In general, the interpretation typically is that a lower cost per programmatic output is better or shows more efficient implementation. However, given the key insurance role that RCD plays, such interpretations are not appropriate and potentially misleading.

### P. Sensitivity analysis and evaluating alternative strategies for implementing RCD

Given that all details and data for this analysis are provided in the additional file, readers are able to evaluate directly how results would change with alternative assumptions on various components of the analysis and to consider alternative strategies for implementation. For example, RCD as implemented in Zambia involves relatively limited use of few high-cost capital goods owned by programme implementers (e.g., mainly vehicles), so total costs are insensitive to the discount rate used in the overall analysis. For example, if the discount rate was increased from 3 to 10 %, the unit cost for training of trainers and CHW costs would increase somewhat as well as unit costs for CHW bicycles and data CHW cell phones (unit cost increases form 7–13 % depending on input), but such unit cost increases would have minor impacts on overall costs.

## Discussion and conclusions

Evaluating the costs of interventions as actually implemented in routine, non-study, settings requires analysts to apply standard methods for evaluating programme costs while adapting analyses based on data available from multiple sources. In many respects, the approach followed here is a variation on prior analyses that model implementation costs prior to actual implementation based on assumed quantities of inputs and estimates of unit costs from various sources (for example, see [37]). The approach presented here, and applied in detail to community malaria case management with reactive case detection, can be applied and adapted as needed to evaluate costs for other interventions such as long-lasting net distribution, indoor residual spraying, or varying forms of mass drug administration.

Evaluating costs of interventions as implemented is substantially more complicated than modelling costs because multiple organizations typically work together during implementation while analysts engaged to complete the costing analysis typically work for one implementing organization or perhaps a separate organization engaged after implementation has begun or completed. If implementing organizations, or perhaps more commonly one or more funders of the implementing organizations, plan to evaluate implementation costs, the earlier in the project cycle the better. In principle, an analysis that models costs prior to implementation can contribute to planning and budgeting (e.g., as outlined in [37]), which can then be assessed following implementation (or an initial implementation period) using the framework outlined here.

While costing analyses often distinguish between recurrent and non-recurrent costs [8, 37], this distinction is generally irrelevant when reporting on the annual cost of implementing an intervention. In addition, while costing analyses often attempt to organize input costs into standard categories (e.g., labour, equipment, transportation), the framework described in this paper develops and presents costs in way that is driven by how the programme is implemented (key activities and stages in the implementation process). While it would be possible to disaggregate each input cost category presented in Table 5 into a generic set of input categories, such detail typically adds little to the overall understanding of costs.

A key contribution of this approach is to identify a primary unit of analysis that is consistent with implementation processes. In the example provided here, the population served by a health facility (the health facility catchment area), served as the primary unit of analysis because community health workers providing services serve their catchment area. With a large number of observations on costs (a total of 164 in the example provided in this paper), the distribution of costs can be assessed and reasons for relatively high and low cost service provision can be identified. This information could be used further to assess further opportunities for efficiency improvements for implementation.

## Additional file

10.1186/s12936-016-1457-5 Costing analysis workbook.

## Abbreviations

ACT: artemisinin-combination therapy
AL: artemether-lumefantrine
CHW: community health worker
CPI: consumer price inflation
dCHW: data community health worker
DHIS: district health information system
DHO: district health office
DMO: district medical officer
DSA: daily subsistence allowance
EHT: environmental health technician
fMDA: focal mass drug administration
GDP: gross domestic product
GRZ: Government of Zambia
HFCA: health facility catchment area
IMF WEO: International Monetary Fund World Economic Outlook
IPT: intermittent preventive treatment
IQR: inter-quartile range
IRS: indoor residual spraying
LLIN: long-lasting insecticidal nets
MACEPA: Malaria Control and Elimination Partnership for Africa
MDA: mass drug administration
NGO: Non-Governmental Organization
NMCC: National Malaria Control Council
RCD: reactive case detection
RDT: rapid diagnostic test
TOT: training of trainers
UNICEF: United Nations Children’s Fund
WHO CHOICE: World Health Organization Choosing Interventions that are cost-effective
ZMW: Zambia Kwacha
